# Genetic variation for body weight change in mice in response to physical exercise

**DOI:** 10.1186/1471-2156-10-58

**Published:** 2009-09-21

**Authors:** Larry J Leamy, Daniel Pomp, J Timothy Lightfoot

**Affiliations:** 1 Department of Biology, University of North Carolina at Charlotte, Charlotte, North Carolina 28223, USA; 2 Department of Kinesiology, University of North Carolina at Charlotte, Charlotte, North Carolina 28223, USA; 3 Departments of Genetics, Nutrition, Cell and Molecular Physiology and the Carolina Center for Genome Science, University of North Carolina, Chapel Hill, NC 27599, USA

## Abstract

**Background:**

Physical activity is beneficial in reducing the weight gain and associated health problems often experienced by individuals as they age, but the association of weight change with physical activity remains complex. We tested for a possible genetic basis for this association between 9-12-week body weight change (WTC) and the distance, duration, and speed voluntarily run by 307 mice in an F_2 _population produced from an intercross of two inbred strains (C57L/J and C3H/HeJ) that differed dramatically in their physical activity levels.

**Results:**

In this population WTC did show the expected negative association with the physical activity traits, but only the phenotypic correlation of WTC with speed (-0.18) reached statistical significance. Using an interval mapping approach with single-nucleotide polymorphism markers, we discovered five (four suggestive and one significant) quantitative trait loci (QTLs) affecting body weight change, only one of which appeared to show pleiotropic effects on the physical activity traits as well. Genome-wide epistasis scans also detected several pairwise interactions of QTLs with pleiotropic effects on WTC and the physical activity traits, but these effects made a significant contribution (51%) only to the covariance of WTC with speed.

**Conclusion:**

It was concluded that the genetic contribution to the phenotypic association between WTC and the physical activity traits in this population of mice was primarily epistatic in origin, restricted to one measure of physical activity, and could be quite variable among different populations depending on the genetic background, experimental design and traits assessed.

## Background

Physical activity has long been considered beneficial in attenuating weight increases that can lead to obesity and associated health problems commonly experienced by many individuals as they age [1-3]. Evidence for this has accumulated from a number of studies, but the relationship between weight change and physical activity remains complex and variable [4-8]. For example, Heitmann et al. [8] showed that the strength of the association between weight changes in monozygotic twins depended on their physical activity levels, being greatest at the highest levels of activity. This finding suggests that like other environmental variables such as nutrition, physical activity may exert its effects by modifying the expression of genes for weight change, especially those alleles that predispose individuals to obesity.

On the other hand, it has become increasingly clear that physical activity itself has a genetic basis, as does the response to exercise as measured by change in body weight [9]. Heritabilities for a variety of different activity measures in humans and rodents typically have been moderate to high in magnitude [7,10-15]. Further, several studies have identified some potential candidate genes in humans [7] that may affect various physical activity traits. It therefore is appropriate to ask whether some of the genes influencing physical activity may also exert pleiotropic effects on weight change. If so, this would suggest that the link between physical activity and weight change is not purely environmental in origin. It also could lead to a better understanding of the considerable variability in weight change typically found among individuals even at the same level of physical activity [7].

Lightfoot et al. [16] and Leamy et al. [17] recently conducted an interval mapping analysis with an F_2 _population of mice generated from an original cross of two inbred strains (C57L/J and C3H/HeJ) that differed in their physical activity levels. These investigators discovered several different quantitative trait loci (QTLs), both single-locus and epistatic, controlling the distance, duration, and speed voluntarily run by the mice during a 21 day interval starting at an average age of 63 days. Leamy et al. [18] followed up this study with an analysis of body weight (at sacrifice) in these mice, and found a number of relationship QTLs that affected one or more of the activity traits variably depending on the phenotypic value of body weight. Body weights were recorded for all the F_2 _mice before and after the testing period, and thus this population presented an opportunity to investigate the genetic association between body weight change (WTC) and physical activity. In the investigation reported here, we first conducted full genome scans to uncover single-locus and epistatic genetic effects on WTC in this population of mice. Using the results of these scans, we then tested for pleiotropic effects of genes and gene interactions common to both WTC and the physical activity traits, and assessed the extent of their impact by the calculation of genetic covariances and correlations.

## Results

Additional file 1 provides basic statistics for weight change and the three physical activity traits used in the analyses. On average during the 3-week testing period, the F_2 _mice gained nearly 2 grams and ran over 6 km each day during a 330 minute span that generated a speed of about 19 meters per minute. WTC ranged from a low of -2.2 g to a high of +6.3 g in these mice, its overall variability being quite high (coefficient of variation = 73). The correlations of WTC with each of the physical activity traits are negative in sign and low in magnitude, being significant only for WTC with speed. The physical activity traits show statistically significant, moderate to high positive correlations. The results of a principal components analysis tend to confirm these trends in the correlations: the first component contrasts weight change with all three physical activity traits, and the second component loads primarily on weight change, emphasizing its independence. The first two principal components account for 85.4% of the total covariance among the four traits.

### Direct-effect QTL scan

**Figure 1:**
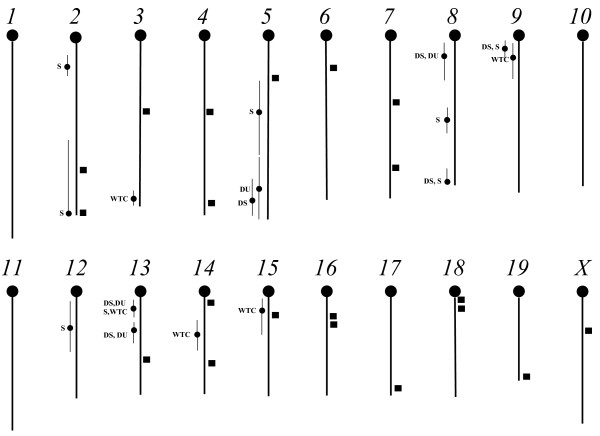
**Locations of direct-effect and epistatic QTLs for WTC and the physical activity traits**. Direct-effect QTLs are shown by circles, epistatic QTLs by squares. DS = distance, DU = duration, S = speed, and WTC = body weight change.

The results of the scan for QTLs affecting weight change are shown in Additional file 2 and are illustrated in Figure 1 (with the locations of other QTLs previously discovered affecting distance, duration, and speed). A total of five QTLs were discovered in this scan, each designated by *WTC *followed by its chromosome number. The LPR scores for four of these QTLs reached the chromosomewise level of significance only whereas that for *WTC3 *was significant at the genomewise level. *WTC13 *affects weight change only in the female mice and *WTC9 *affects weight change only in males.

All five QTLs for weight change exhibit significant additive genotypic effects, their absolute values averaging 0.29 standard deviations (Additional file 2). The positive sign of the *a *value for *WTC3 *means that the C57L/J allele at this locus tends to increase the amount of change in body weight (CC mean WTC = 2.39 g) more so than the C3H/HeJ allele (HH mean WTC = 1.75 g). The reverse is true for the other four QTLs, however, suggesting that the allele from the higher activity C57L/J strain acts to decrease body weight (reduce WTC) during the 3-week test period. Dominance genotypic values average 0.26 standard deviations, although only one value (for *WTC9*_*M*_) reaches significance. Individually, the QTLs contribute from 4.2 to 7.7% of the total variance of weight change. The collective contribution of single-locus effects for these QTLs, estimated by the adjusted *R*^2 ^value from a multiple regression analysis, is 13% (average of separate sexes).

### Epistasis Scan

The epistasis genome scan produced 63 QTL combinations for weight change that reached significance at the 1% level. A chi-square test confirmed that this number was significantly (*P *< 0.01) greater than the 30.3 expected by chance alone. This result also corresponds to a false discovery rate of 30.3/63, or 0.48, suggesting that roughly one-half of these 63 putative instances of epistasis are likely to be real.

At the 0.1% significance level, ten pairwise combinations of QTLs showed epistasis for weight change (Additional file 3), this being greater (*P *< 0.01) than the 3.0 combinations expected by chance alone (FDR = 3.0/10 = 30%). The probabilities associated with the *F *tests for epistasis range from 0.000020 to 0.000946. Six of these probabilities reach the suggestive, although none reach the significant, 0.05 Bonferroni threshold level. The QTLs in these ten pairs occur on 14 of the 20 chromosomes (see also Figure 1), with six chromosomes involved in two combinations. QTLs in specific regions on chromosome 16 (18--22 cM) and chromosome 18 (4--10 cM) appear to be particularly important because they participate in the three epistatic combinations that exhibit the lowest probabilities. Only one epistatic QTL on chromosome 15 appears to colocalize with a QTL (*WTC15*; Additional file 2) exhibiting direct effects on WTC.

Among the 10 QTL combinations, a total of 18 epistatic components reached significance at the conventional 5% level (Additional file 3). Three of the 10 pairwise QTL combinations exhibited only one significant epistatic component whereas the remaining 7 exhibited two or more. The means of the (absolute) values of the *aa*, *ad*, *da*, and *dd *components, respectively, are 0.24, 0.65, 0.37, and 0.27 standard deviations. The *ad *components clearly average much higher than the others, and in fact 9 of these 10 values are statistically significant. Thus the significant *ad *values comprise one-half of the 18 total significant epistatic components, but the distribution of these *aa*, *ad*/*da*, and *dd *values (4, 13, 1) still follows the expected 1:2:1 ratio (*P *> 0.05 in a chi-square test).

We assessed the total impact of epistasis on the variability of weight change via a multiple regression model using the 18 significant epistatic components associated with the 10 QTL pairs. Whereas the regression of WTC on the significant additive and index values for the three direct-effect QTLs found to be significant showed they contributed 13% (adjusted *R*^2^) of the total variation, addition of the epistatic components to this model increased the adjusted *R*^2 ^value to 43%. This increase is statistically significant (*P *< 0.01), and suggests that epistatic effects of just these 10 QTL combinations are sufficient to account for 30% of the total variability of weight change.

### WTC Associations

To assess the genetic association of WTC with the physical activity traits, we searched for evidence for pleiotropy from both direct-effect and epistatic QTLs. For the direct-effect QTLs, only *WTC9*_*M *_and *WTC13*_*F *_mapped fairly close to any of the QTLs for the physical activity traits, so we conducted pleiotropy tests using these QTLs. The tests yielded a positive result only for *WTC13*_*F*_, suggesting that this QTL may be the same as one previously found in this location affecting distance, duration, and speed [16]. This putative common QTL has a negative additive genetic effect on WTC (Additional file 2), but a positive additive genetic effect on all three physical activity traits [16]. For the combined sexes, however, none of the three weight change QTLs colocalize with QTLs for the physical activity traits (Figure 1). In terms of direct effects, therefore, the genetic basis for body weight gain in the presence of exercise appears to be generally distinct from that for the physical activity traits themselves in these mice.

Additional file 4 summarizes the results of the additional canonical correlation analyses performed to search for epistatic pleiotropy for WTC with each of the three physical activity traits. The number of epistatic combinations reaching the 0.001 probability level for the pairs of traits was relatively restricted: 2 for WTC with distance, 4 for WTC with duration, and 5 for WTC with speed. In these analyses, one QTL combination reached the 5% suggestive, and two reached the significant, Bonferroni threshold level of significance. The individual epistatic components that reach significance also are shown in the table, and in general most appear to be different for WTC compared to the physical activity trait. Positive and negative components are roughly equal, although all those for the 5 QTL pairs affecting speed are negative in sign.

**Figure 2:**
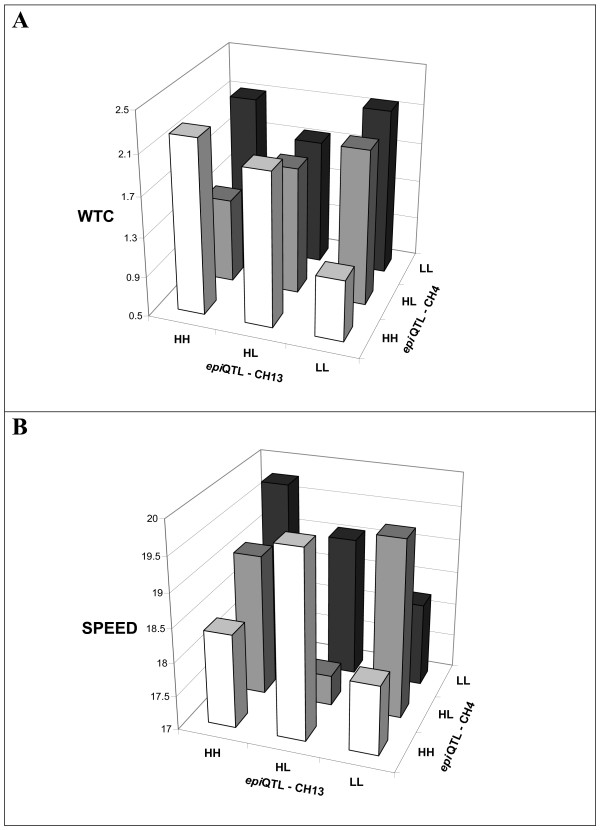
**Epistatic interactions between QTLs on chromosomes 4 and 13**. (A) Effects on weight change, (B) effects on speed. HH = C3H/C3H homozygotes, HL = C3H/C57L heterozygotes, and LL = C57L/C57L homozygotes.

To illustrate epistatic pleiotropy, we plotted the genotypic means of the two epistatic QTLs on chromosomes 4 and 13 affecting both WTC and speed (see Figure 2). These plots clearly show that this particular pair of QTLs has very different effects on these two traits. Thus for WTC, the additive effect (difference between homozygotes) of the QTL on chromosome 13 is large for the HH and HL genotypes of the QTL on chromosome 14 but small for the LL genotype whereas the precise reverse is true for speed. Dominance effects (under- versus overdominance) also are generally different for the two traits. In general, the net epistatic effects tend to generate a negative correlation between the two traits.

We calculated phenotypic, epistatic genetic, and environmental correlations of WTC with each of the physical activity traits in the combined sexes (see Methods for the approach used). Single-locus genetic correlations were not calculated because single-locus pleiotropic effects were detected only for one QTL in females. As may be seen in Additional file 5, phenotypic correlations of WTC with distance, duration, and speed are negative in sign and low in magnitude, although those for distance and speed reach statistical significance. These values differ somewhat from those given previously in Additional file 1 (calculated from phenotypic data) because as explained in Methods, we used only those individuals with complete SNP data. Environmental correlations for these three combinations all are negative in sign, but none quite reach significance after sequential Bonferroni adjustment. Epistatic genetic correlations also all are negative, and are higher in magnitude than the phenotypic correlations, although only that for WTC with speed is statistically significant. Epistatic genetic contributions to the phenotypic correlations average slightly over 1/3, with the remainder being environmental in origin.

## Discussion

### Genetic architecture of WTC

The basic intent of this study was to test for a genetic (pleiotropic) association between WTC and each of the three physical activity traits in our population of mice. For this purpose, we first assessed the genetic architecture of WTC by conducting genome scans to uncover single-locus and epistatic QTLs affecting this trait. It will be recalled that we discovered five QTLs in the single-locus scan, two of which were sex-specific. This number of QTLs for WTC seems relatively low, but only in comparison to the number found in other studies [19,20] for weight change in mice not engaged in wheel running or other similar physical activities. Our measure of WTC assessed the amount of weight change experienced by the mice during a 3-week period of physical activity, so such comparisons are not entirely appropriate.

The QTLs we found for WTC presumably represent areas of the genome affecting the amount of change in body weight in the mice in response to physical activity. They were uncovered in our F_2 _population of mice, the progenitors of which were selected on the basis of their divergence in physical activity traits [16], not body weight or weight gain. Body weights at the time of testing were very similar for both the C57L/J (mean = 22.4 g) and C3H/HeJ (23.0 g) inbred strains [16]. We therefore would not necessarily expect these QTLs to generally correspond to any of the well-known growth or body weight QTLs discovered in mice. And in fact none of the five QTLs for WTC map in the same area as a number of QTLs found for late-age weight gain in two studies using F_2 _mice derived from lines selected for divergence in body weight [19] or growth [20].

The five QTLs for WTC cumulatively explained about 13% of the total variation in this trait. All showed significant additive genotypic effects, with *WTC9*_*M *_also exhibiting significant dominance effects (Additional file 2). If we recalculate the joint contribution of these QTLs using only the additive genotypic effects, this yields a QTL heritability for WTC of 0.11. This value is quite low, reflecting the few QTLs for WTC we discovered segregating in this F_2 _population of mice. Heritability estimates for weight change (or gain) in mice not voluntarily exercising also have tended to be low, varying from 0 to about 0.2 or 0.3 for weight change (or gain) in other mouse populations [19-22]. Heritabilities also have been estimated as 0.15 for adult life body weight change [23] and 0.24 for long-term weight change [24] from participants in the Framingham Heart Study.

In contrast to direct effects of QTLs, epistatic interactions of QTLs appeared to exert much more of an impact on WTC in our population of mice, contributing 30% of the total variation in this trait. This estimate may be too high, however, since it was derived using the 10 QTL pairs reaching significance at the 0.1% level (at least three of which were predicted to be false positives). Recalculation of the epistatic impact using those 6 pairs reaching the 0.05 suggestive threshold level (see Additional file 3) in fact reduces the contribution to 17%, although this is still more than that from direct-effect QTLs. On the other hand, none of the epistatic interactions reaching significance at the 5% or 1% level were used in this calculation, some of which surely represent real instances of epistasis. So although the true contribution of epistasis on WTC cannot be known, it seems reasonable to suggest that it is greater, perhaps considerably so, than that due to single locus effects.

It was interesting that only one direct-effect QTL for WTC (*WTC15*, see Additional file 2) colocalized with any of the QTLs epistatically affecting WTC, suggesting it has both direct and indirect effects on this trait. QTLs on chromosomes 16 and 18 exerted particularly strong epistatic effects on WTC yet no direct-effect QTLs were found on either of these chromosomes. In part this may be a consequence both of the degree of statistical power inherent in our epistasis tests and our restriction of the epistatic interactions to those reaching significance only at the stringent 0.1% level. However, it is not uncommon to find that many or most QTLs participating in epistatic interactions affecting various traits are different from those directly affecting the traits [17,25].

The low narrow-sense heritability and the substantial epistatic genetic contribution for WTC fit the profile of major fitness traits [26,27]. Such traits include litter size and maternal performance, for example, that Peripato et al. [28,29] showed were significantly affected by only two individual QTLs but by a number of epistatic QTL combinations. It is possible that we underestimated the number of QTLs with direct effects on WTC, and thus its heritability, especially given the variability of this trait and the modest size of the F_2 _population. On the other hand, perhaps this trait has limited genetic variability. Additional studies clearly are needed before we can discover how general this finding might be and whether this trait truly is an important component of fitness.

### WTC Associations

The relatively few QTLs discovered for WTC exhibited very little overlap in their positions in the genome with those for the physical activity traits [16], suggesting a general independence, at least in terms of direct effects, of the QTLs for WTC versus those for the physical activity traits. Only one QTL (on chromosome 13) was identified as potentially common to all traits, and this must be viewed with caution because of the limited power of the pleiotropy tests. Further, this QTL affected the physical activity traits in both sexes [16] but WTC in females only. While there is no immediate explanation for this difference, it is not surprising given the nearly independent genetic architecture for WTC versus that for the physical activity traits.

If we suppose that the QTL on chromosome 13 is truly common, it is suggestive that it exhibited antagonistic pleiotropy. That is, the C57L/J allele at this locus acted to increase distance, duration, and speed [16,18] while decreasing WTC (the reverse would be true for the C3H/HeJ allele), precisely in the direction that might be expected. This sort of pattern is reminiscent of the action of *NHLH2 *that has been shown to reduce physical activity in mice and thereby increase body weight that eventually leads to adult-onset obesity [30]. Even if there are QTLs throughout the genome with these sorts of antagonistic pleiotropic effects, however, our results suggest that they play at best a very minor role in contributing to the association of WTC with physical activity.

Instead, as evidenced from our joint epistasis scans, the genetic contribution to the WTC-physical activity association appears to be primarily epistatic in origin. Thus these scans showed 2-5 pairwise interactions of QTLs significantly (*P *< 0.001) affecting both WTC and each of the physical activity traits (recall Additional file 4). Originally 7 to 11 QTL combinations showed overall significance for both traits, but using our criterion for epistatic pleiotropy, we reported only those instances in which both WTC and the activity trait individually reached significance. As was the case with the epistasis scan for WTC alone, some of these may be false instances of epistasis, although we may place more confidence in the three QTL combinations that reached the suggestive genomewise significance threshold. Again, presumably there are other QTL combinations that represent true instances of epistasis jointly affecting WTC and the physical activity traits that were not included because we restricted our analysis to those reaching the 0.1% significance level.

Whatever the identity of the epistatic QTLs jointly affecting WTC and the physical activity traits (Additional file 4), none colocalized with the five direct-effect QTLs for WTC (Additional file 2) or with any of those previously found for distance, duration, and speed [16]. There is, however, some overlap with pairwise QTL interactions found to affect WTC (see Additional file 3) and those previously found for the physical activity traits [17]. Specifically, an interaction of QTLs on chromosome 5 and 19 affecting duration and WTC individually also jointly affected both WTC and duration, and another interaction of QTLs on chromosomes 2 and 7 affecting speed individually also jointly affected speed and WTC. But these are just two out of 11 total epistatic interactions with joint effects (Additional file 4), suggesting that the QTLs with epistatic pleiotropic effects on WTC and physical activity are generally distinct from those individually affecting either trait.

It is useful to note that epistatic pleiotropy may occur for several traits with little or no impact on the covariances of these traits. Wolf et al. [31] have shown that in an orthogonal model such as that used here, the contribution of epistatic pleiotropy to the covariance of two traits depends on the patterns of epistasis exhibited by these traits. To impact covariances, both traits must show significance for the same epistatic component(s) (*aa*, *ad*, *da*, or *dd*) since these components are independent. Further, common epistatic components with the same sign will generate a positive covariance whereas if they differ in sign, a negative covariance will result [31]. From an inspection of the epistatic pleiotropic effects detailed in Additional file 4, therefore, we would predict that epistasis would make little contribution to the covariance between WTC and either distance or duration (none of the 6 combinations share a common, significant epistatic component). But since WTC and speed share three common, significant epistatic components, two of which are different in sign, we would expect epistatic pleiotropy to generate a substantial (negative) covariance between these two traits.

These expectations generally were borne out by the magnitude of covariances and correlations of WTC with each of the physical activity traits previously presented (Additional file 5). Epistatic pleiotropic effects contributed on average about 26% of the phenotypic covariance of WTC with distance and duration when little was expected, but the epistatic genetic correlations were non-significant and thus this contribution could have resulted simply from sampling variation. On the other hand, epistasis accounted for 50% of the phenotypic covariation between WTC and speed, and generated a highly significant, negative epistatic genetic correlation (-0.56). The square of this correlation suggests that WTC shares about 31% of its epistatic genetic variance with speed. Among the three physical activity measures, therefore, WTC appears to possess a genetic association only with speed.

While there may be a significant contribution of epistatic pleiotropy to the phenotypic covariance between WTC and speed, the phenotypic association itself between these two traits is rather weak. Thus the phenotypic correlation between WTC and speed of only -0.18 suggests that speed accounts for no more than about 3% of the phenotypic variation in WTC. Calculation of this correlation among mice with varying levels of speed, however, reveals an interesting trend. For mice in the lowest quartile, middle two quartiles, and highest quartile of the distribution of speed, respectively, Spearman's correlation of WTC with speed is -0.31 (*P *< 0.01), -0.09 (*P *= 0.29) and +0.11 (*P *= 0.36). Weight loss is therefore greatest for mice in the lowest speed quartile as they increase their physical activity, but becomes less effective for mice at median levels of speed and especially for individuals predisposed to run at the highest speeds.

This finding is not surprising given the well-known phenomena in humans whereby the higher the intensity of exercise, the less free fatty acids are used to support ATP production [32]. Thus, the mice active at higher speeds would be less likely to further decrease body weight due to their higher intensity of exercise. Conversely, it is also well accepted that at moderate and low exercise intensities, the contribution of free-fatty acids to support ATP production can range from 50-80% depending on several factors, thus inducing weight loss in those mice that are doing activity at lower speeds [32]. Whatever the explanation, however, this trend is independent of overall body size since the mean body weights at the start (and end) of the test period in the low, intermediate, and high speed groups are not significantly different (*P *> 0.05 in one-way ANOVAs).

The non-significant phenotypic correlations of WTC with both distance and duration was somewhat surprising. This suggests that at least for the three-week test period, the distance or duration the mice ran was completely independent of their amount of weight change. Both correlations were negative in sign as might have been expected, however, so perhaps with a lengthened period of testing they might have increased in magnitude and reached significance. Whatever the case, our results suggest that among these three physical activity traits, speed shows the greatest association with weight change.

It should be emphasized that our results as discussed are applicable strictly to mice that were measured during a relatively short time period (average of 9- to 12-weeks of age) that is roughly equivalent to early adulthood in humans. We chose this time interval because in mice this is the age when physical activity is the highest and is fairly stable [33,34]. Before this time, the mice are still increasing in activity whereas after 12 weeks, most of them begin a long, steady decline in activity [33,34]. Thus, this three week period is sufficient to provide good average measures of the physical activity traits.

It is difficult to say how our results might have changed had these mice been measured at older ages or for a longer testing period. We did perform some preliminary analyses on weight change during the entire (6-week) period during which weights were taken for each mouse, and discovered five QTLs affecting this 6-week weight change trait. Two of these QTLs were on chromosomes 9 and 13 at similar locations to those found for WTC, but others were found on chromosomes 1, 7, and 8. This suggests that the genetic architecture of weight change can certainly change depending on both the age and period of time it is measured, and thus we might expect its associations with the physical activity traits to change as well.

## Conclusion

It seems clear that weight change in mice from our F_2 _population was very nearly independent of the level of physical activity they exhibited during the 3-week test period. All three measures of activity showed a negative phenotypic correlation with WTC as might be expected, but these correlations were low in magnitude and only that for speed was clearly statistically significant. We have shown that there is some genetic basis for the WTC-speed association that it is primarily due to epistatic rather than direct effects of QTLs. These estimates of epistatic contributions, however, are strictly applicable only to our F_2 _population with its assumed equal frequencies of alleles at each locus and orthogonality of the epistatic components. As allele frequencies change and generate non-independent epistatic components, the contribution of epistasis to the covariances of traits would also be expected to change [35]. Thus in other populations the epistatic contribution to the WTC-speed covariance may well be greater or lesser than we found, and would be created anew each generation through the normal segregation of alleles. So even if we knew the genotypes of all appropriate epistatic loci in a population, our results suggest that this would be of limited use in predicting the amount of weight change (loss) expected for a given level of physical activity. We must look to other genetic and environmental factors and discover how they interact with physical activity to more fully understand the large degree of inter-individual variability we typically see in the impact of physical activity on weight change.

## Methods

### The population and traits

An F_2 _population of mice was generated from crossing two inbred strains, C57L/J (CC) and C3H/HeJ (HH), chosen because of their divergence in physical activity measures. Reciprocal crosses of these inbreds produced F_1 _offspring that in turn were crossed and eventually produced 310 F_2 _offspring. All mice were housed in a single room in the University of North Carolina at Charlotte Vivarium maintained at 18-21°C and 20-40% humidity with 12 hour light/dark cycles. Food (Harland Teklad 8604 Rodent Diet, Madison, WI) and water were provided *ad libitum*. In the rearing and testing of these mice, we followed all guidelines approved by the UNC Charlotte IACUC, the American Physiological Society, and the American College of Sports Medicine.

After weaning at 21-28 days, the mice were individually housed in rat cages mounted with a solid surface running wheel on which was glued a magnetic sensor. For each mouse, this sensor counted the number of total wheel revolutions and time spent exercising [16]. These data generated three physical activity traits: total daily distance run in kilometers, total daily exercise duration in minutes, and average daily running speed in meters/minute.

The F_2 _mice were measured for each of these three physical activity traits during a 21 day interval and daily values were averaged to account for any fluctuations over this time period. The age of the mice at the start of testing averaged 63 days (9-weeks), varying from 46 to 76 days (24 mice were 46-53 days of age, 227 mice were 57-64 days of age, and 56 mice were 71-76 days of age). Body weights also were recorded weekly, and for each mouse, weight change (WTC) was calculated as the difference in body weight at the start and the end of the testing period.

All mice were sacrificed after completion of the physical activity measurements, and had their kidneys harvested for subsequent DNA extraction. For the interval mapping analysis of traits described below, all F_2 _mice were genotyped at 129 single-nucleotide polymorphisms (SNPs) that differed between the C57L/J and C3H/HeJ progenitor strains. These SNPs provided good coverage of the entire genome with an average intermarker interval of about 14 cM.

For the analysis of weight change, 307 total mice were available. We first plotted WTC in these mice and verified that its distribution was normal (using the Kolmorogov-Smirnov test and the 0.01 probability level). We also adjusted WTC (and distance, duration, and speed) for significant effects due to sex, litter size, and rearing block (but not age at testing since this factor did not reach significance). This was done by calculating residuals from a linear model in which these three variables were treated as classification factors [16].

Once these data were adjusted, we first calculated basic statistics for WTC and the three physical activity traits in these 307 mice to summarize their central tendency and variability. To discover trends in the associations among these traits, we also calculated all pairwise correlations and performed a principal components analysis. For the subsequent genetic analyses, it was necessary to combine the phenotypic and the genetic data. Since there were some missing genotypic (SNP) data, however, estimates of QTL effects and associations of traits (see below) were based on sample sizes that were reduced some from 307, and that varied among the genotypes.

### Direct-effect QTL analyses

We used the regression approach of interval mapping [36] to search for direct-effect QTLs for WTC. This analysis was accomplished using canonical correlation analyses at every location 2 cM apart on each chromosome, with WTC as the single variable in one group, and additive and dominance index values in the other group [16]. These analyses generated Wilks Lambda statistics with their associated probabilities that were logarithmically transformed to calculate LPR values [(log_10_(1/Prob.)] similar to LOD scores [37]. We considered the highest LPR score on each chromosome to indicate a putative QTL at that location if this score exceeded a threshold value. We used the permutation method of Churchill and Doerge [38] with 1000 shuffles of the phenotypic data to estimate both 5% threshold values for each chromosome that were suggestive of linkage as well as a 5% genome-wise threshold value that offered significant evidence of linkage. Confidence intervals for each QTL were determined by the one-LOD rule [39]. Each chromosome was also tested for two-QTLs and sex-specific QTLs affecting weight change in the manner already described [16].

We used multiple regression of body weight gain on the additive and dominance index values of the QTLs discovered for WTC to estimate their additive (*a*) and dominance (*d*) genotypic values and to test these values for significance. The *a *values estimate one-half of the difference between the mean WTC values of the two homozygotes and the *d *values estimate the difference between the mean WTC of the heterozygotes and that of the mean of the two homozygotes [26]. The multiple regression analyses also generated coefficients of determination that were useful in assessing the individual and collective impact of the QTLs on weight gain.

### Epistatic QTL analyses

We conducted a genome scan for epistatic QTLs affecting WTC via canonical correlation analyses for all pairs of locations on each of the 190 total possible pairwise combinations of the 20 chromosomes (no attempt was made to evaluate epistasis among loci within chromosomes). Each analysis used WTC as the variable in one group, but all four interactions of the additive and dominance genotypic scores from the two chromosomes as the variables in the other group, while partialling the main effects associated with these scores. We plotted all probabilities generated from these analyses that were less than 0.05 and assumed those pairs of positions exhibiting the lowest probability within the probability valleys were potential QTL combinations exhibiting epistasis. Where these plots yielded clearly separated valleys of probabilities, each valley was taken as a separate instance of epistasis [25].

To determine the extent of significant epistasis given the multiple comparisons problem inherent in the hundreds of tests run, we used the effective marker approach outlined by Li and Ji [40]. To implement this approach, we conducted principal components analyses of the correlation matrices of the genotypic values at all loci 2 cM apart on each chromosome to estimate the number of independent (SNP) markers. We then were able to calculate the total number of independent epistasis tests by the sum of the crossproducts of the effective number of markers for all 190 pairs of chromosomes. This sum was 3032, suggesting that we might expect about 152 tests to be significant at the 5% level, 30.3 at the 1% level, and 3.0 at the 0.1% level because of chance alone. Significant epistasis for WTC therefore was indicated if the observed number of tests reaching a given probability level significantly exceeded its expected number (using a chi-square statistic associated with 1 d.f.). At the various probability levels, the expected number of epistatic tests also was divided into the total number actually found to be significant to estimate the false discovery rate, or FDR [41].

We also tested for individual instances of epistasis by calculating the 0.05 Bonferroni threshold level of significance as 0.05/3032 = 1.65 × 10^-5^. Thus any probability in an individual epistatic test less than this value was considered as *significant *evidence for epistasis [29,42]. This Bonferroni threshold level is considered rather stringent, however, so we also estimated a more liberal threshold level using the number of chromosome pairs, as suggested by Holland [43]. This yielded a threshold probability of 0.05/190 = 2.63 × 10^-4 ^that if reached by any individual test was considered to be *suggestive *of epistasis.

For those QTL pairs exhibiting probabilities less than 0.001 in the overall epistasis tests, we conducted multiple regression analyses to estimate additive by additive (*aa*), additive by dominance (*ad*), dominance by additive (*da*), and dominance by dominance (*dd*) genotypic epistatic effects. The *aa *type of epistasis for two loci (A and B) occurs when the *a *value at one locus (A) differs depending on what genotype (*B*/*B*, *B*/*b*, or *b*/*b*) is present at another locus and *vice versa*. The *ad *epistatic type occurs when the *a *value for a locus A differs depending on the genotype at another locus B whereas the *d *value at B differs depending on the locus A genotype (and *vice versa *for dominance by additive epistasis). Finally, *dd *epistasis occurs when the *d *value at locus A differs depending on the genotype at locus B and *vice versa *[44]. Tests for the significance of each of these four genotypic epistasis terms were done via individual *t*-tests using the conventional 5% probability level. The multiple regression procedure also was useful in assessing the total impact of epistasis on WTC.

### Genetic associations of WTC with the activity traits

Once all putative direct-effect and epistatic QTLs for WTC were discovered and their effects estimated, we were interested in assessing the extent of the genetic association between WTC and the physical activity traits produced from pleiotropy of common genes. To this end, we first inspected the locations of all direct-effect QTLs found for WTC to see if any colocalized with QTLs for distance, duration, or speed. Where this was the case, we conducted pleiotropy tests using the procedure outlined by Knott and Haley [45] that involves a comparison of the determinants of two matrices and generates a likelihood-ratio (chi-square) test statistic. If this statistic was significant, it suggested that the QTLs were in separate locations whereas a non-significant result suggested that there could be a common QTL exerting pleiotropic effects on both WTC and one or more of the physical activity traits.

To test for QTL interactions influencing multiple traits (epistatic pleiotropy), we ran three additional epistasis scans. These were conducted as described above using canonical correlation, but this time for WTC with each of the physical activity traits (i.e., WTC with distance, WTC with duration, and WTC with speed). Once each of these scans was completed, we followed the same procedure as already described for the epistasis analysis of WTC alone. For all QTL combinations reaching significance at the 0.001 probability level, we used a multiple regression approach to test for overall significance of epistasis for each of the two traits (WTC and the physical activity trait) and for the significance of the individual epistatic components. If both traits showed overall epistatic significance for a given pair of QTLs, it was assumed that these QTLs were exhibiting epistatic pleiotropy.

Once all QTLs pleiotropically affecting WTC and one or more of the physical activity traits were located, it was possible to assess the genetic and environmental contributions to the phenotypic integration among these traits [31]. To accomplish this, we computed phenotypic, single-locus genetic, epistatic genetic, and environmental correlations of WTC with each of the three physical activity traits. Phenotypic correlations were calculated for the traits not adjusted in any way, and environmental correlations were calculated from the residuals of a regression of each trait on the single-locus and epistatic components that reached statistical significance in the pleiotropy tests. We also subtracted these residuals from the corresponding phenotypic covariances to calculate single-locus and epistatic covariances and variances. All covariances were converted to correlations by dividing them by the square root of the product of the two appropriate variances. To assess the impact of the genetic and environmental sources of covariation to the phenotypic covariances, we calculated the percent of the phenotypic covariance explained by each of these covariances. Finally, we generated 1000 bootstrap samples of the data to estimate standard errors for each of the correlations and to test them for significance. In evaluating significance, we used the sequential Bonferroni procedure [46] for the three estimates in each of the categories of correlations.

## Authors' contributions

LJL performed the data analysis, wrote and prepared the manuscript for submission. JTL was the principal supervisor of the study and assisted with preparation of the manuscript. DP designed the genome scan including SNP selection, and reviewed the manuscript. All authors read and approved the final manuscript.

## Supplementary Material

Additional file 1 **Basic statistics for weight change and the physical activity traits. ** Click here for file

Additional file 2 **QTLs affecting weight change**. Click here for file

Additional file 3 ** Epistatic QTLs affecting WTC.** Click here for file

Additional file 4 ** Epistatic QTLs with overall significant effects on WTC and the physical activity traits.** Click here for file

Additional file 5 ** Phenotypic, genetic and environmental correlations of WTC with the physical activity traits.** Click here for file

## References

[B1] Schmitz KH, Jacobs DR, Leon AS, Schreiner PJ, Sternfeld B (2000). Physical activity and body weight: associations over ten years in the CARDIA study. International Journal of Obesity.

[B2] Sternfeld B, Wang H, Quesenberry P, Abrams B, Everson-Rose SA, Greendale GA, Matthews KA, Torrens JI, Sowers M (2004). Physical activity and changes in weight and waist circumference in midlife women: findings from the study of women's health across the nation. American Journal of Epidemiology.

[B3] Basterra-Gortari FJ, Bes-Rastrollo M, Pardo-Rernandez M, Forga L, Martinez JA, Martinez-Gonzalez MA (2009). Changes in weight and physical activity over two years. Spanish Alumni Medicine & Science in Sports & Exercise.

[B4] Kirk EP, Jacobsen DJ, Givson C, Hill JO, Donnelly JE (2003). Time course for changes in aerobic capacity and body composition in overweight men and women in response to long-term exercise: the Midwest Exercise Trial (MET). International Journal of Obesity.

[B5] Yoshioka M, Doucet E, St-Pierre S, Almeras N, Richard D, Labrie A, Despres JP, Bouchard C, Tremblay A (2001). Impact of high-intensity exercise on energy expenditure, lipid oxidation and body fatness. International Journal of Obesity.

[B6] Donnelly JE, Jacobsen DJ, Heelan KS, Seip R, Smith S (2000). The effects of 18 months of intermittent vs continuous exercise on aerobic capacity, body weight and composition, and metabolic fitness in previously sedentary, moderately obese females. International Journal of Obesity.

[B7] Teran-Garcia M, Rankinen T, Bouchard C (2008). Genes, exercise, growth, and the sedentary, obese child. Journal of Applied Physiology.

[B8] Heitmann BL, Kaprio J, Harris JR, Rissanen A, Korkeila M, Koskenvuo M (1997). Are genetic determinants of weight gain modified by leisure-time physical activity? A prospective study of Finnish twins. American Journal of Clinical Nutrition.

[B9] Nehrenberg DL, Hua K, Estrada-Smith D, Garland T, Pomp D Voluntary exercise and its effects on body composition depend on genetic selection history. Obesity.

[B10] Kaprio JM, Koskenvuo M, Sarna S, Gedda L, Parisi P, Nance WE (1981). Cigarette smoking, use of alcohol and leisure-time activity among same-sexed adult male twins. Progress in Clinical and Biological Research Twin Research 3: Epidemiological and Clinical Studies.

[B11] Perusse L, Tremblay A, LeBlanc C, Bouchard C (1989). Genetic and environmental influences on level of habitual physical activity and exercise participation. American Journal of Epidemiology.

[B12] Lauderdale DS, Fabsitz R, Meyer JM, Sholinsky P, Ramakrishnan V, Goldberg J (1997). Familial determinants of moderate and intense physical activity: a twin study. Medicine & Science in Sports & Exercise.

[B13] Houle-Leroy P, Garland T, Swallow JG, Guderley H (2000). Effects of voluntary activity and genetic selection on muscle metabolic capacities in house mice *Mus domesticus*. Journal of Applied Physiology.

[B14] Rhodes JS, Garland T (2003). Differential sensitivity to acute administration of Ritalin, apomorphine, SCH 23390, but not raclopride in mice selectively bred for hyperactive wheel-running behavior. Psychopharmacology.

[B15] Lightfoot JT, Turner MJ, Daves M, Vordermark A, Kleeberger SR (2004). Genetic influence on daily wheel running activity level. Physiological Genomics.

[B16] Lightfoot TL, Turner MJ, Pomp D, Kleeberger SR, Leamy LJ (2008). Quantitative trait loci for physical activity traits in mice. Physiological Genomics.

[B17] Leamy LJ, Pomp D, Lightfoot JT (2008). An epistatic genetic basis for physical activity traits in mice. Journal of Heredity.

[B18] Leamy LJ, Pomp D, Lightfoot JT (2009). Genetic variation in the pleiotropic association between physical activity and body weight in mice. Genetics Selection Evolution.

[B19] Vaughn T, Pletscher L, Peripato A, King-Ellison K, Adams E, Erikson C, Cheverud J (1999). Mapping quantitative trait loci for murine growth - A closer look at genetic architecture. Genetical Research.

[B20] Rocha JL, Eisen EJ, Van Vleck D, Pomp D (2004). A large-sample QTL study in mice: I. Growth. Mammalian Genome.

[B21] Jara-Almonte M, White JM (1973). Genetic relationships among milk yield, growth, feed intake and efficiency in laboratory mice. Journal of Animal Science.

[B22] Clutter AC, Pomp D, Murray DJ (1996). Quantitative genetics of transgenic mice: Components of phenotypic variation in body weights and weight gains. Genetics.

[B23] Golla A, Strauch K, Dietter J, Baur MP (2003). Quantitative trait linkage analysis of longitudinal change in body weight. BMC Genetics.

[B24] Fox CS, Heard-Costa NL, Ramachandran SV, Murabito JM, D'Agostino RB, Atwood LD (2005). Genomewide linkage analysis of weight change in the Framingham Heart Study. The Journal of Endrocrinology & Metabolism.

[B25] Leamy LJ, Workman MS, Routman EJ, Cheverud JM (2005). An epistatic genetic basis for fluctuating asymmetry of tooth size and shape in mice. Heredity.

[B26] Falconer DS, Mackay TFC (1996). Introduction to Quantitative Genetics.

[B27] Malmberg RL, Mauricio R (2005). QTL-based evidence for the role of epistasis in evolution. Genetical Research.

[B28] Peripato AC, de Brito RA, Matioli SR, Pletscher LS, Vaughn TT, Cheverud JM (2004). Epistasis affecting litter size in mice. Journal of Evolutionary Biology.

[B29] Peripato AC, de Brito RA, Vaughn TT, Pletscher LS, Matioli SR, Cheverud JM (2002). Quantitative trait loci for maternal performance for offspring survival in mice. Genetics.

[B30] Good DJ, Coyle CA, Fox DL (2008). Nhlh2: a basic helix-loop-helix transcription factor controlling physical activity. Exercise and Sport Sciences Reviews.

[B31] Wolf JB, Leamy LJ, Routman EJ, Cheverud JM (2005). Epistatic pleiotropy and the genetic architecture of covariation within early and late-developing skull trait complexes in mice. Genetics.

[B32] Powers SK, Howley ET (2009). Exercise Metabolism. Exercise Physiology: Theory and Application to Fitness and Performance.

[B33] Turner MJ, Kleeberger SR, Lightfoot JT (2005). The influence of genetic background on daily running-wheel activity differs with aging. Physiological Genomics.

[B34] Swallow JG, Garland T, Carter PA, Zhan WZ, Sieck GC (1998). Effects of voluntary activity and genetic selection on aerobic capacity in house mice (*Mus domesticus*). Journal of Applied Physiology.

[B35] Cheverud J, Routman E (1995). Epistasis and its contribution to genetic variance components. Genetics.

[B36] Haley CS, Knott SA (1992). A simple regression technique for mapping quantitative trait loci in line crosses using flanking markers. Heredity.

[B37] Lander ES, Botstein D (1989). Mapping mendelian factors underlying quantitative traits using RFLP linkage maps. Genetics.

[B38] Churchill GA, Doerge RW (1994). Empirical threshold values for quantitative trait mapping. Genetics.

[B39] Lynch M, Walsh B (1998). Genetics and Analysis of Quantitative Traits.

[B40] Li J, Ji L (2005). Adjusting multiple testing in multilocus analyses using the eigenvalues of a correlation matrix. Heredity.

[B41] Storey JD, Tibshirani R (2003). Statistical significance for genome-wide studies. Proceedings of the National Academy of Sciences.

[B42] Leamy LJ, Routman EJ, Cheverud JM (2002). An epistatic genetic basis for fluctuating asymmetry of mandible size in mice. Evolution.

[B43] Holland JB (1998). EPISTACY: a SAS program for detecting two-locus epistatic interactions using genetic marker information. Journal of Heredity.

[B44] Cheverud JM, Wolf J, Brodie II E, Wade M (2000). Detecting epistasis among quantitative trait loci. Epistasis and the Evolutionary Process.

[B45] Knott SA, Haley CS (2000). Multitrait least squares for quantitative trait loci detection. Genetics.

[B46] Rice WR (1989). Analyzing tables of statistical tests. Evolution.

