# Supplementary material for: Genetic variation for body weight change in mice in response to physical exercise
Source: BMC Genet. 2009 Sep 21;10:58. doi: 10.1186/1471-2156-10-58 (PMC2760581; doi:10.1186/1471-2156-10-58)
Supplement: Additional file 1 — Basic statistics for weight change and the physical activity traits. [file 1471-2156-10-58-S1.pdf]

Table 1

Basic statistics for weight change and the physical activity traits

			Components		Correlations		
	Mean	Std Dev	I	II	Distance	Duration	Speed
Weight Change (g)	1.84	1.35	-0.12	0.98	-0.09	-0.05	-0.15*
Distance (km/day)	6.36	2.39	0.63	0.10		0.92**	0.71**
Duration min/day)	330.41	101.00	0.57	0.16			0.45**
Speed meters/min)	18.70	3.42	0.51	-0.08			

Shown are means and standard deviations for body weight change, distance, duration, and speed in the 307 F_2 mice. Also shown are loadings on the first two components (I and II) derived from a principal components analysis, and pairwise correlations among these four traits. * = $P < 0.05$; ** = $P < 0.01$
