# Supplementary material for: Genetic variation for body weight change in mice in response to physical exercise
Source: BMC Genet. 2009 Sep 21;10:58. doi: 10.1186/1471-2156-10-58 (PMC2760581; doi:10.1186/1471-2156-10-58)
Supplement: Additional file 2 — QTLs affecting weight change. [file 1471-2156-10-58-S2.pdf]

Table 2

QTLs affecting weight change

Sex	QTL	Ch	Marker	Marker Dist	Cent. Dist	CI	LPR	%	*a/SD*	*d/SD*
Males	*WTC3*	3	*gnf01.149.507*	12	89	81—99	2.86	10.30	0.32**	-0.49**
	WTC9	9	*rs13480073*	14	15	5—41	2.78	8.61	-0.16	0.54**
	WTC15	15	*CEL-15_52357711*	0	20	10—32	2.70	8.93	-0.24*	-0.47**
Females	*WTC13*	13	*rs6329684*	8	9	1—37	2.66	7.85	-0.41**	0.23
	WTC14	14	*CEL-14_115843331*	0	62	58—62	2.57	8.23	-0.22*	-0.47**

Shown are locations, confidence intervals (CI), LPR scores ($\log_{10}Prob^{-1}$), percentage of the variation explained (%), and standardized additive (*a*) and dominance genotypic values (*d*) for QTLs on all chromosomes (Ch) significantly affecting weight change. QTLs are designated *WTC* followed by their chromosome number and a subscript if they affect only males (*M*) or only females (*F*). Locations are given as map distances from the nearest proximal marker (Marker Dist) and from the centromere (Cent. Dist), and confidence intervals are expressed as distances from the centromere. All LPR values are significant at the 5% chromosomewise level or at the genomewise level (†).
* = $P < 0.05$; ** = $P < 0.01$.
