# Supplementary material for: Genetic variation for body weight change in mice in response to physical exercise
Source: BMC Genet. 2009 Sep 21;10:58. doi: 10.1186/1471-2156-10-58 (PMC2760581; doi:10.1186/1471-2156-10-58)
Supplement: Additional file 3 — Epistatic QTLs affecting WTC. [file 1471-2156-10-58-S3.pdf]

Table 3

Epistatic QTLs affecting WTC

CH1	cM	CH2	cM	*F*	Prob. *F*	*aa*	*ad*	*da*	*dd*
2	83	14	48	5.57	**0.000252**	-0.24	0.79**	0.35	0.06
2	100	15	14	4.79	0.000946	-0.11	-0.83**	-0.41	0.44
3	47	17	64	4.95	0.000718	-0.15	0.61**	-0.50**	0.08
4	47	7	50	5.08	0.000579	0.32*	0.47**	0.46*	0.22
4	98	13	43	4.86	0.000838	0.37*	0.22	0.67**	0.24
5	28	19	53	5.59	**0.000243**	0.00	-0.89**	-0.23	0.19
6	21	X	29	5.65	**0.000221**	-0.47**	-0.63*	0.15	0.39
7	80	18	10	6.22	**0.000083**	0.08	-0.79**	-0.22	0.62*
14	6	16	22	6.64	**0.000041**	0.49**	0.67**	-0.18	-0.15
16	18	18	4	7.06	**0.000020**	0.14	0.62**	0.57**	-0.32

Shown are locations in cM from the centromere for QTLs on each pair of chromosomes (CH1 and CH2) whose *F* tests for overall epistasis affecting weight change resulted in a probability less than 0.001 (probabilities in bold reach the 0.05 suggestive Bonferroni threshold level) . For each pairwise QTL combination, the individual standardized epistatic components also are given. * = P < 0.05; ** = P < 0.01
