# Supplementary material for: Genetic variation for body weight change in mice in response to physical exercise
Source: BMC Genet. 2009 Sep 21;10:58. doi: 10.1186/1471-2156-10-58 (PMC2760581; doi:10.1186/1471-2156-10-58)
Supplement: Additional file 4 — Epistatic QTLs with overall significant effects on WTC and the physical activity traits. [file 1471-2156-10-58-S4.pdf]

Table 4

Epistatic QTLs with overall significant effects on WTC and the physical activity traits

						WTC				Activity			
CH1	cM	CH2	cM	*F*	Prob. *F*	*aa*	*ad*	*da*	*dd*	*aa*	*ad*	*da*	*dd*
										Distance			
5	35	19	55	5.35	**0.0000017**	0.05	-0.71**	-0.27	-0.02	-0.17	0.29	-0.34	-0.81**
16	33	17	65	3.51	0.0005699	0.15	-0.29	0.58**	0.22	0.40**	-0.03	-0.35	0.11
										Duration			
3	68	6	91	3.40	0.0007965	-0.37*	-0.03	-0.25	-0.81*	-0.25	0.80**	-0.16	-0.31
5	35	19	55	5.31	**0.0000020**	0.05	-0.71**	-0.27	-0.02	-0.20	0.20	-0.40*	0.77
7	42	11	50	3.35	0.0009265	-0.22	0.19	-0.54**	-0.31	-0.19	0.41*	-0.36	-0.40
9	48	10	78	3.42	0.0007526	0.01	-0.52**	0.43**	-0.00	0.28*	0.34	-0.24	0.17
										Speed			
2	75	7	38	3.70	0.0003174	0.15	-0.19	-0.43*	0.53*	-0.23*	0.18	0.30	-0.77**
3	62	7	68	3.75	0.0002727	0.22	0.06	-0.72**	0.19	-0.45**	-0.01	0.39	-0.32
4	105	13	67	3.79	**0.0002409**	0.64**	0.20	0.17	-0.32	0.19	-1.08**	-0.07	-0.20
5	11	14	64	3.42	0.0007540	-0.11	0.07	0.75**	0.04	-0.15	-0.20	-0.58**	-0.43
9	86	11	88	3.42	0.0007489	-0.20	-0.50*	0.13	0.38	-0.06	-0.59**	-0.08	-0.50

Shown are locations in cM from the centromere for QTLs on each pair of chromosomes (CH1 and CH2) whose *F* tests for overall epistasis for weight change (WTC) with each of the physical activity traits resulted in a probability less than 0.001 (probabilities in bold reach the 0.05 suggestive Bonferroni threshold level). For each pairwise QTL combination, standardized individual epistatic components also are given. * = $P < 0.05$; ** = $P < 0.01$.
