# Supplementary material for: Genetic variation for body weight change in mice in response to physical exercise
Source: BMC Genet. 2009 Sep 21;10:58. doi: 10.1186/1471-2156-10-58 (PMC2760581; doi:10.1186/1471-2156-10-58)
Supplement: Additional file 5 — Phenotypic, genetic and environmental correlations of WTC with the physical activity traits. [file 1471-2156-10-58-S5.pdf]

Table 5

Phenotypic, genetic and environmental correlations of WTC with the physical activity traits

	Phenotypic	Epistatic		Environmental	
Traits	r_P	r_I	%	r_E	%
WTC-Distance	-0.12*	-0.50	(34.1)	-0.09	(65.9)
WTC-Duration	-0.07	-0.09	(17.4)	-0.07	(82.6)
WTC-Speed	-0.18**	-0.56**	(51.1)	-0.11	(48.9)
Means	-0.12	-0.39	(34.2)	-0.08	(65.8)

Shown are phenotypic (r_p), epistatic or interaction genetic (r_I), and environmental correlations (r_E) of WTC with each of the physical activity traits in the F_2 mice. The percentage of the phenotypic covariance between traits explained by each of these sources of covariation also is given in parentheses. * = $P < 0.05$; ** = $P < 0.01$
